# Expression of cancer cell-intrinsic PD-1 associates with PD-L1 and p-S6 and predicts a good prognosis in nasopharyngeal carcinoma

**DOI:** 10.7150/jca.60739

**Published:** 2021-08-24

**Authors:** Yuting Zhang, Xianyong Chen, Hongmei Zheng, Yuting Zhan, Jiadi Luo, Yang Yang, Yue Ning, Haihua Wang, Weiyuan Wang, Songqing Fan

**Affiliations:** 1 Department of Pathology, the Second Xiangya Hospital, Central South University, Changsha, Hunan, 410011, China.; 2 Department of Pathology, Affiliated Hospital of Xiangnan University, Chenzhou City, Hunan, 423000, China.; 3 Department of Pathology, Xiangya Hospital of Central South University, Changsha, 410011, Hunan, China.

**Keywords:** nasopharyngeal carcinoma, cancer cell-intrinsic PD-1, PD-L1, p-S6, prognosis

## Abstract

**Aims:** Programmed cell death ligand 1 (PD-L1) is the ligand of programmed death 1 (PD-1), which is a host immunity inhibitory receptor. Expression of PD-L1 in diverse tumor types has been widely discussed, while there is little research about tumor intrinsic-PD-1. Phospho-S6 (p-S6) is an important downstream effector in the PI3K/AKT/mTOR pathway. Our study was focused on investigating expression of cancer cell-intrinsic PD-1, PD-L1 and p-S6 proteins and aimed to illustrate their relationship and clinical significances in nasopharyngeal carcinoma (NPC).

**Methods:** The expression of PD-1, PD-L1 and p-S6 proteins in tissues of NPC, non-cancerous nasopharyngeal epithelia, primary cancer and matching metastatic lesion was detected by immunohistochemistry.

**Results:** Expression of PD-1, PD-L1 and p-S6 proteins and co-expression of PD-1 and PD-L1 were significantly higher in NPC (all *P*<0.05). The expression of PD-1 and co-expression of PD-1 and PD-L1 in paired metastatic NPC were significantly increased (all *P*<0.01). NPC patients with positive expression of PD-L1 showed significantly higher overall survival rate (*P* =0.035). However, NPC patients with positive expression PD-1 and p-S6 showed significantly lower overall survival rate (*P* =0.031, *P*=0.044, respectively). Interestingly, NPC patients with co-expression of PD-1 and PD-L1 had lower overall survival rate (*P*=0.042). Multivariate Cox proportional hazard regression analysis confirmed that positive expression of PD-L1 and p-S6 were independent prognostic factors for NPC patients.

**Conclusions:** Expression of cancer cell-intrinsic PD-1 associates with PD-L1 and p-S6 proteins, PD-L1 might serve as a good prognostic biomarker, while p-S6 could be an independent poor prognostic biomarker for NPC patients.

## Introduction

Nasopharyngeal carcinoma (NPC) is one of the most common cancers in Asia, especially in southern China 1. Epstein-Barr virus (EBV) is a recognized cause of NPC. Infection, genetic susceptibility, high nitrite food and smoking are independent risk factors for NPC 2. Nowadays, treatments of NPC are mainly radiotherapy and chemotherapy. Patients with early-stage of NPC could benefit from radiotherapy or/and chemotherapy, thereby obtaining a longer survival time [3_,_ 4]. Unfortunately, most NPC patients, at the time of diagnosis, are already in the advanced stage, and chemotherapy, radiotherapy, targeted therapy or immune treatment still cannot significantly effectively extend the survival time of them. 5-7. In recent years, studies have found that immune escape of tumor cells and abnormal activation of signaling pathways play important roles in the occurrence and development of NPC 1. Finding new targets of NPC will provide new clues for exploring more effective treatment of NPC patients.

Programmed death 1 (PD-1), a cell membrane protein with 288 amino acids, is a protein from the CD28 superfamily. The expression of tumor cell-intrinsic programmed death 1 (PD-1) played an important role in melanoma tumorigenesis 8. As one of major ligands of PD-1, programmed death ligand 1 (PD-L1) also takes part in the tumor progression 9. Ribosomal protein S6 (S6) could be activated by phosphorylated p70S6K which is a downstream effector of the AKT/mTOR pathway. Activated S6 is related to poor prognosis of NPC via the messenger RNA translation machinery 10-11. Recent research found that the cancer cell-intrinsic PD-1 activated by PD-L1 would promote phosphorylation of S6 and, initiate the translation process, which played a vital role in tumor occurrence, development, invasion and metastasis 12-13. However, whether there is abnormal activation of the cell-intrinsic PD-1/PD-L1 axis in NPC, and the relationship between it and S6/p-S6 expression have not been studied yet.

In this study, we evaluated expression of cancer cell-intrinsic PD-1, PD-L1 and p-S6 proteins in 281 cases of NPC and 51 cases of non-cancerous nasopharyngeal epithelia, as well as in 24 primary NPC and their matched metastases, to illustrate the relationship between expression of cancer cell-intrinsic PD-1, PD-L1 and p-S6 proteins and their clinical significances in NPC.

## Methods

### Dataset Download

KM Plotter (http://kmplot.com/analysis/index.php?p=service&cancer=pancancer_rnaseq) was used to detect the prognostic value of PD1, PD-L1 and S6 in head-neck squamous cell carcinoma.

### Ethics Statement

All experimental protocols were approved by the Ethics Review Board of the Second Xiangya Hospital, Central South University (Scientific and Research Ethics Committee, No: Y202/2014), and informed consent was applied to all samples.

### Tissue samples and clinical data

All samples were paraffin-embedded tissue, including 281 NPC tissues, 24 primary NPC and their matched metastases, and 51 non-cancerous control nasopharyngeal epithelia, from Department of pathology, the Second Xiangya Hospital of Central South University during January 2008 to December 2017. All clinical record and the follow-up data were obtained. These cases were pathologically diagnosed and classified according to the latest WHO (February 2017) stage category of head and neck tumors. NPC patients in the present study did not receive radiotherapy or chemotherapy prior to biopsy. The time period from first diagnosis to the date of death or the last known date alive was defined as the overall survival time. All NPC samples were divided into different clinically stages according to the standard in UJCC/AJCC staging system 14,15. Epstein-Barr virus encoded RNAs (EBER) were detected by *in-situ* hybridization. Characteristics of patient were presented in supplementary Table S1.

### Immunohistochemistry and scores

PD-1, PD-L1 and p-S6 proteins staining was employed by ready-to-use Max Vision TM^+^ HRP-Polymer anti-Mouse IHC Kit (Dako; Carpintrria, CA) on 4 μm tissue sections. As described in our previous publication 11, 1:100 dilution of primary antibody to PD-1 (Mouse polyclonal antibody, Catalog #MX033, MXB Biotechnologies, China), PD-L1 (Rabbit monoclonal antibody, Catalog #ab228462, Abcam, UK), and Phospho-S6 (p-S6) ^Ser235/236^ (Rabbit polyclonal antibody, Catalog #4857, Cell Signaling Technology, USA) were used to detected the expression of those three proteins in all samples. Each experiment included positive and negative control slide. To confirm the specificity of the antibody, we used the matched IgG isotype antibody as a negative control.

Immunohistochemical staining was independently evaluated under a light microscope at a magnification of ×200 by Y Zhang and Y Zhan blinded to patients' information. The score calculation method was: cancer cell-intrinsic PD-1 16 was assessed as positive for NPC cells with a greater than 5 positive percentage. PD-L1 17 scored NPC by calculating tumor positive scores (TPS). The TPS criterion is the ratio of the sum of positive tumor cells relative to total tumor cells. PD-L1 was regarded as positive when the score was higher than 5. Staining scores ≥2 was considered as positive expression for an optimal cut-off value for p-S6 18. The two reviewers scored a concordance rate of 95%, and the discordance were resolved by looking at microscopic slides and discussion again.

### Statistical analysis

The relationship between expression of cancer cell-intrinsic PD-1, PD-L1, p-S6 and PD-L1/PD-1 proteins and clinicopathological features in NPC was analyzed using chi-square test. The pairwise association between PD-1, PD-L1, p-S6 and PD-1/PD-L1 expression in NPC was approached through the Spearman's rank correlation coefficient. Kaplan‑Meier analysis was hired to draw the overall survival curves, and the log‑rank test was the tool to evaluate statistical significance. Cox comparative hazards model was performed to assess the independent prognostic factors of NPC with PD-1, PD-L1, p-S6 and PD-1/PD-L1 expression. All the above analysis was completed by SPSS (IBM SPSS Statistics 24.0) software. Based on two-sided statistical analysis, *P* < 0.05 was considered to be statistically significant.

## Results

### Association between expression of cancer cell-intrinsic PD-1, PD-L1, p-S6, and co-expression of PD-1 and PD-L1 and clinicopathological features in NPC

**Figure 1:**
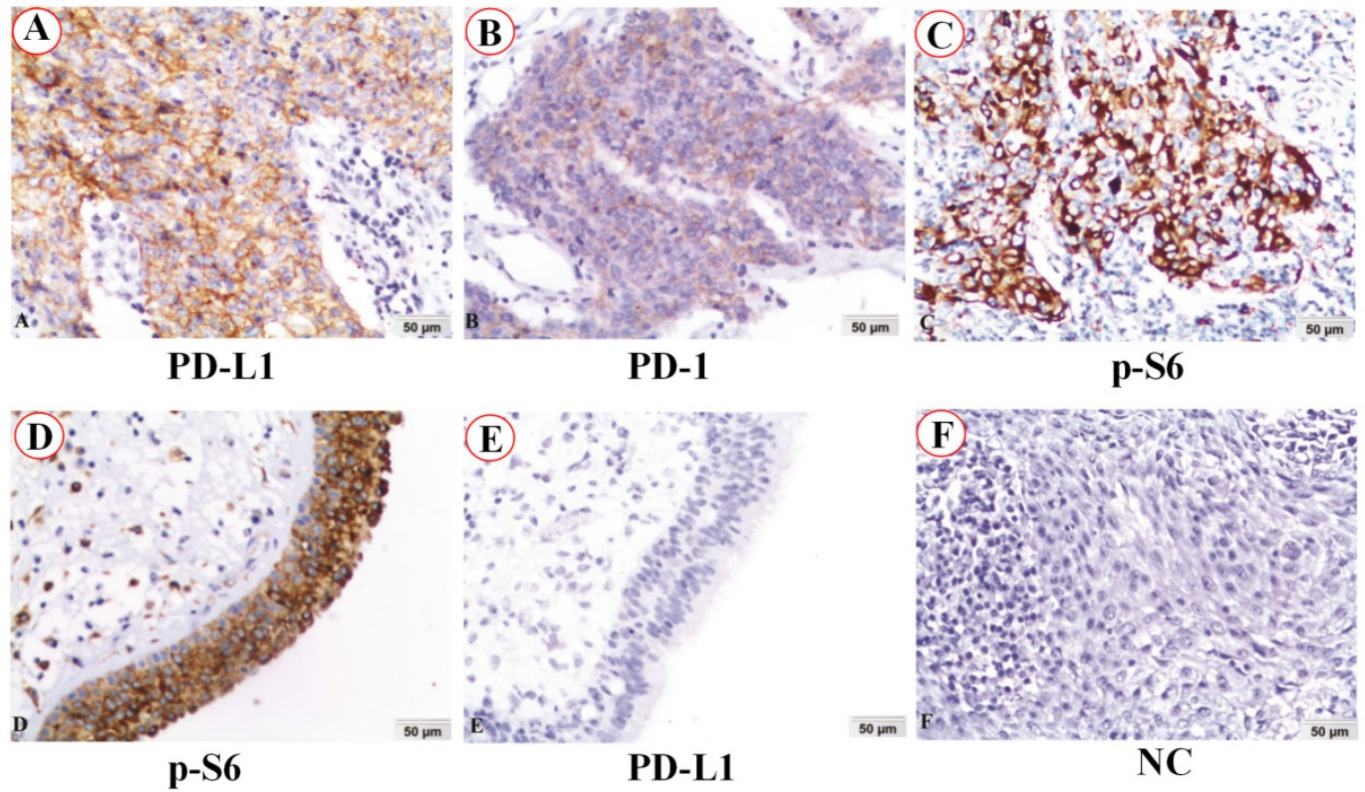
PD-L1, PD-1 and p-S6 proteins expression in NPC and non-cancerous nasopharyngeal epithelia was detected using immunohistochemistry (IHC). A: PD-L1 protein staining was located in the membrane of NPC. B: PD-1 protein staining was in the cytoplasm of NPC. C and D: Staining of p-S6 protein was discovered in the cytoplasm in both NPC and the control normal nasopharyngeal epithelia. E: No staining of PD-L1 protein was found in the control normal nasopharyngeal epithelia. F: No staining showed up in negative control (NC) in the NPC (IHC, DAB staining, x200).

The expression and subcellular localization of PD-L1, PD-1 and p-S6 proteins in NPC and non-cancerous control nasopharyngeal epithelia were detected by immunohistochemistry (IHC). PD-L1 protein staining was located in the membrane of NPC (Figure 1A), while PD-1 protein staining was in the cytoplasm of NPC (Figure 1B). Staining of p-S6 protein was discovered in the cytoplasm in both NPC (Figure 1C) and the control normal nasopharyngeal epithelia (Figure 1D). No staining of PD-L1 protein was in the normal nasopharyngeal epithelia (Figure 1E). No staining showed up in negative control in the NPC (Figure 1F) (IHC, DAB staining, ×200).

**Figure 2:**
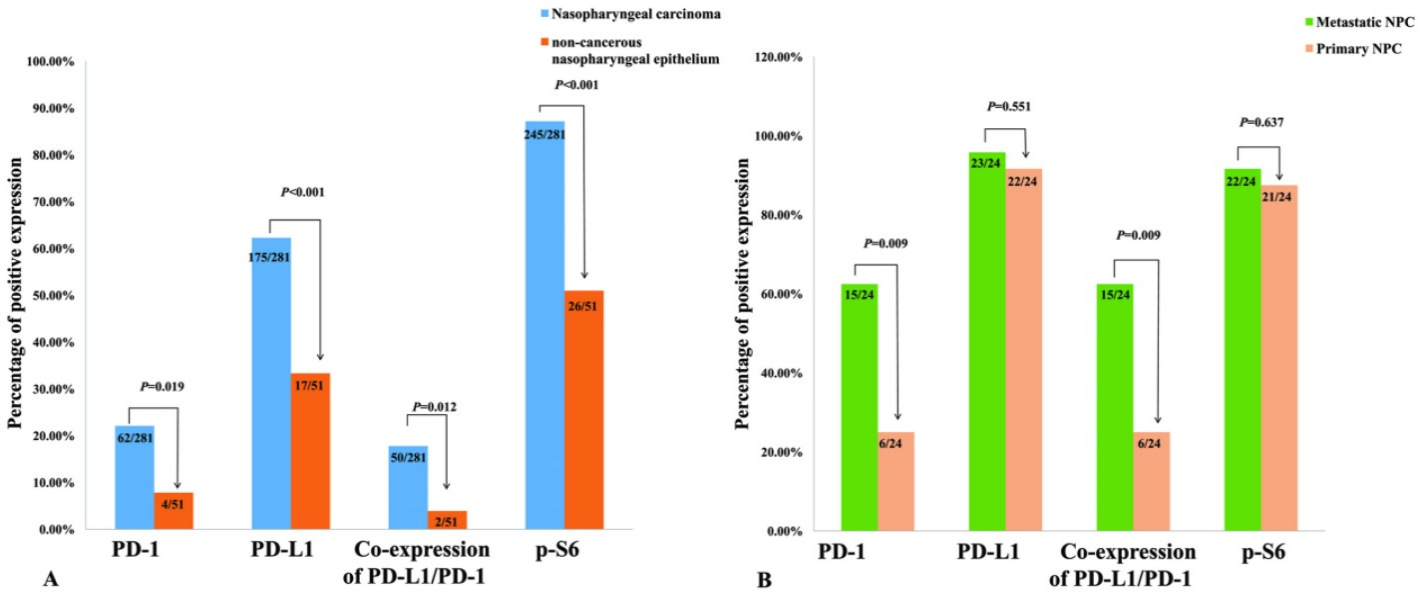
Positive expression of PD-1, PD-L1, p-S6 and common expression PD-1/PD-L1 in 281 NPC and 51 non-cancerous control nasopharyngeal epithelia, 24 pairs of primary NPCs and their corresponding metastatic lesion were analyzed by χ^2^ test. **A:** The percentages of positive expression of PD-1, PD-L1, p-S6 and co-expression PD-1/PD-L1 in NPC were evidently higher than those in the non-cancerous nasopharyngeal epithelia (all* P*<0.05). **B:** There were significantly lower expression of PD-1 and co-expression of PD-1/PD-L1 in primary NPC compared to the matched metastatic lesion (all* P*<0.01).

The positive expression of cancer cell-intrinsic PD-1, PD-L1, p-S6 and co-expression of PD-1 and PD-L1 in NPC was 22.1% (62/281), 62.3% (175/281), 87.2% (245/281), and 17.8% (50/281), respectively. However, the positive expression of these proteins in non-cancerous nasopharyngeal epithelia was dramatically lower 7.8% (4/51), 33.3% (17/51), 51.0% (26/51) and 3.9 (2/51), respectively (all *P*<0.05) (Figure 2A).

We further investigated expression of cancer cell-intrinsic PD-1, PD-L1, p-S6 and co-expression of PD-1 and PD-L1 in the primary NPC and their matched lymph node metastatic lesion. Results in Figure 2B showed that the positive percentage of PD-1 expression in the primary NPC (25.0%, 6/24) was significantly lower than that in matched metastasis (62.5%, 15/24) (*P*=0.009), as well as the co-expression of PD-1 and PD-L1 (*P*=0.009) (Figure 2B). It's worth noting that in these primary and matched metastatic samples, all samples with PD-1 positive also acquired PD-L1 positive. No obvious difference in the expression of PD-L1 and p-S6 protein between primary NPC and their matched metastasis yet (*P*> 0.05).

We then explored the relationship between cancer cell-intrinsic PD-1, PD-L1, p-S6 and co-expression of PD-L1/PD-1 proteins and clinicopathological features of NPC patients including gender, age, clinical T/N/M category, clinical stages, histological type and lymph node metastasis status. These results were displayed in Table 1. The positive percentage of PD-L1 (*P* =0.002) was statistically lower in NPC patients with clinical T1 category than those in T2, T3 and T4, but the patients with clinical N0, N1 and N2 category was evidently higher than that clinical N3 (*P* =0.015). However, the positive percentages of PD-1 and co-expression of PD-L1 and PD-1, and p-S6 were not associated with gender, clinical N or M category, clinical stages, histological type and lymph node status (all *P*> 0.05).

### Correlations of cancer cell-intrinsic PD-1, PD-L1, p-S6 expression and co-expression of PD-L1 and PD-1 in NPC

Furthermore, we investigated whether there were some correlations among these proteins. The relationship between PD-1, PD-L1, p-S6 expression and co-expression of PD-L1/PD-1 proteins in 281 NPC patients was shown in Table 2. PD-L1 expression was positively associated with PD-1 (r = 0.219, *P* <0.001), p-S6 (r = 0.273, *P* < 0.001) or co-expression of PD-L1 and PD-1 (r=0.366, *P* <0.001) in NPC. In addition, p-S6 was also positively related to PD-1 (r=0.127, *P*=0.033) and co-expression of PD-L1 and PD-1 in NPC (r=0.153, *P*=0.01). Consistent with the notable phenomenon, expression of cancer cell-intrinsic PD-1 was strongly related to combined PD-L1 and PD-1 expression (r=0.885, *P*<0.001).

### The impact of expression of cancer cell-intrinsic PD-1, PD-L1, p-S6, and co-expression of PD-L1 and PD-1 proteins on prognosis in NPC patients

**Figure 3:**
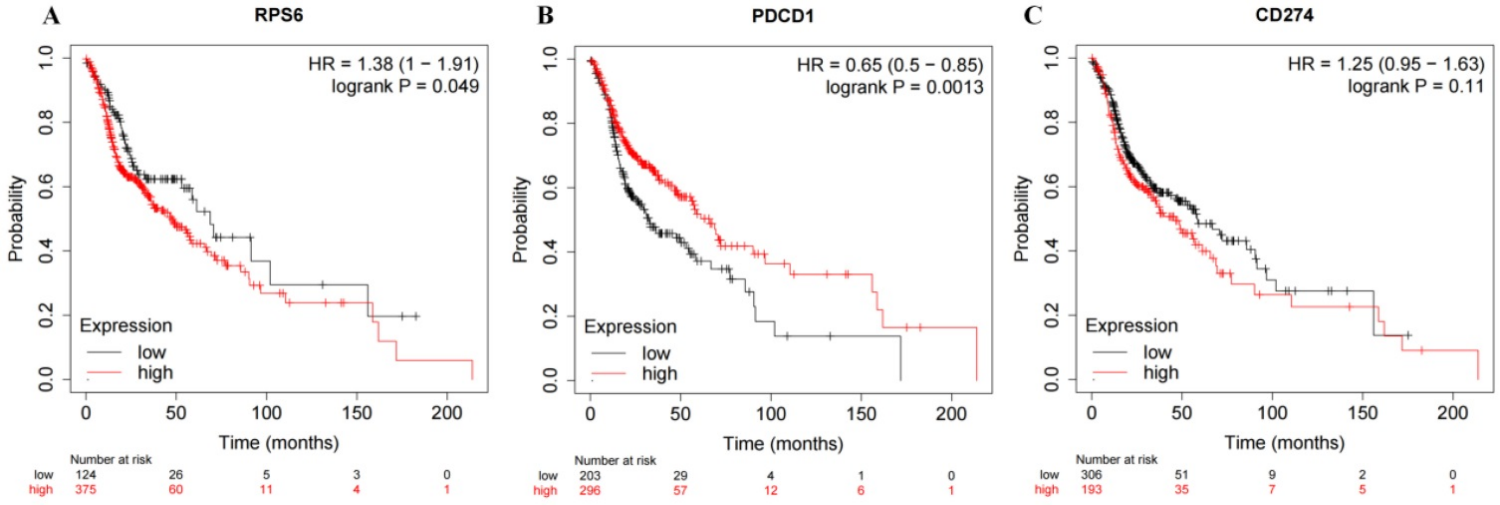
Bioinformatics analysis. **A:** K-M survival analysis of RPS6 (S6) mRNA expression. **B:** K-M survival analysis of PDCD1 (PD1) mRNA expression. **C:** K-M survival analysis of CD274 (PD-L1) mRNA expression.

KM-Plotter was used to predict the prognostic value of PD-1, PD-L1 and S6 mRNA expression. Head and neck squamous cell carcinoma patients with low expression of RPS6 (S6) and high expression of PDCD1 (PD1) had good prognosis (Figure 3; both *P*<0.05). However, no significantly prognostic value was seen in CD274 (PD-L1, *P>*0.05).

**Figure 4:**
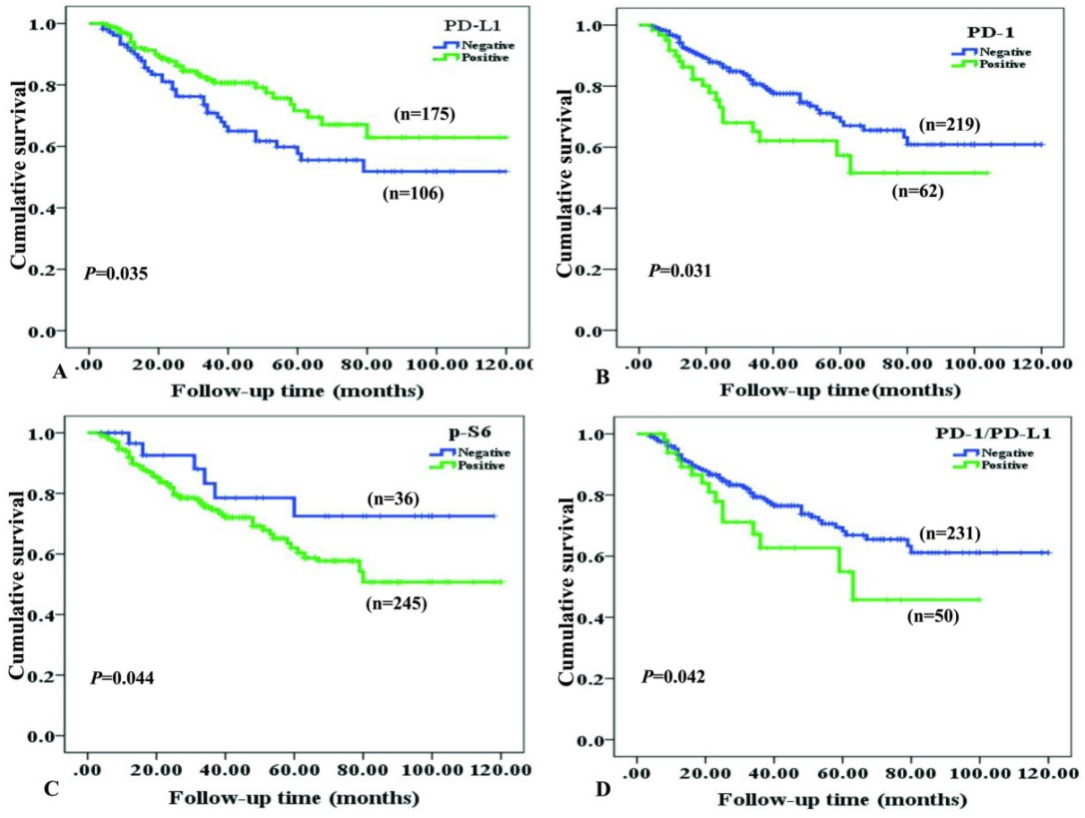
Kaplan-Meier curves for overall survival of NPC patients with PD-L1, PD-1, p-S6 proteins expression and co-expression of PD-L1/PD-1. **A:** NPC patients with positive expression of PD-L1 caught a longer OS (overall survival) (*P*=0.035); **B:** NPC patients with negative PD-1 staining showed a longer OS (*P*=0.031); **C:** NPC patients with negative p-S6 revealed a longer OS (*P*=0.044); D: NPC patients with common positive expression of PD-L1/PD-1 had a shorter OS (*P*=0.042).

Survival situation of NPC patients with differentially expressed cancer cell-intrinsic PD-1, PD-L1, p-S6 and co-expression PD-L1 and PD-1 proteins was studied through Kaplan-Meier survival curves (Figure 4). As to NPC patients, overall survival rate was significantly higher in cases with positive expression of PD-L1, compared to ones with negative PD-L1 expression (*P*=0.035, Figure 4A). On the contrary, NPC patients with positive expression of PD-1 (*P*=0.031, Figure 4B), p-S6 (*P* =0.044, Figure 4C) or co-expression of PD-1 and PD-L1 (*P*=0.042, Figure 4D) had shorter survival time than others by univariate analysis.

Furthermore, we investigated whether cancer cell-intrinsic PD-1, PD-L1, p-S6 and co-expression of PD-L1 and PD-1 proteins could be used as independent prognostic factors for NPC patients. Data in Table 3 revealed that positive expression of PD-L1 protein was identified as an independent good prognostic factor (*P*=0.002), while positive expression of p-S6 protein (*P*=0.003), lymph node metastasis (LNM) status (*P*=0.004), clinical N category (*P*<0.001), M category (*P*< 0.001) and clinical stages (*P*=0.005) were identified as independent poor prognostic factors for overall survival of NPC patients. However, patients with positive PD-1 expression and co-expression of PD-L1 and PD-1 had no significant impact on the overall survival of NPC patients (*P* >0.05, respectively). Other factors including gender, age, histological type and T/N stage category also have no obvious impacts on the prognosis in NPC (all *P* > 0.05).

## Discussion

As a ligand for PD-1, PD-L1 is a transmembrane protein expressed on immune cells and tumor cells encoded by the CD274 gene. It is not only a cancer-promoting factor in some certain malignant tumors, such as gastric cancer and esophageal cancer, also as protective factor in some other tumors including Merkel cell carcinoma and breast cancer 19-24. However, the roles of PD-L1 action in NPC is not clear. It has been reported that short overall survival time of NPC patients was related to high expression of PD-L1 25-26. But other research results drew an opposite conclusion that patients with high PD-L1 expression obtained a better prognosis 27-29, which was in line with our results. We found that there was significant higher positive expression of PD-L1, PD-1, p-S6 and combined PD-1 and PD-L1 in NPC than that of in non-cancerous nasopharyngeal epithelia. Furthermore, PD-L1 expression had effects on T-stage category of NPC. In conclusion, overexpression of PD-1, PD-L1, combined PD-L1 and PD-1 and p-S6 were all related with tumorigenesis.

Cox multivariate regression analysis showed that positive expression of PD-L1 in NPC patients had a lower death risk. In fact, the association between the expression of PD-L1 and tumor immune evasion is not directly proportional, and may just represent the persistence of anti-tumor response 30. Here's more evidence that PD-L1 could take part in the regulation of anti-tumor immunity. It was found that AhR (Aryl hydrocarbon receptor) activation could mediate the expression of PD-1 in CD8^+^ T cells. Maybe their co-stimulators AhR induced the correlation expression between PD-1 and combined PD-L1 and PD-1 expression 31-32. The secretion of interferon gamma (IFNγ) and the enhancement of tumor PD-L1 expression promote each other, resulting in CD8^+^ and CD3^+^ T cells enrichment around NPC tissues, and the accumulation of these immune cells promotes the immune response to tumor tissue 30, 33-34.

As ligands of PD-1, PD-L1 and PD-L2 are mostly found in tumor cells and antigen presenting cells. And they will inhibit the tumor cell death through down-regulating the T cell response when they bind to PD-1. The transmembrane receptor PD-1 is a prominent checkpoint primarily expressing on T cells 35-36. PD-1 plays vital roles in promoting NPC growth and is related to the short overall survival time in NPC patients 37. Studies focused on PD-1 protein in NPC have primarily examined PD-1 expression in lymphocytes 38-39. Kleffel's team firstly reported that PD-1 protein was also presented in cancer cells and promoted tumor occurrence and development in melanoma 8. Other studies have also shown cell-intrinsic PD-1 promotes the development of liver cancer and pancreatic cancer, and shortens the survival rate 16, 40. Tumor cell-intrinsic PD-1 promotes tumor occurrence such as hepatocellular carcinoma and melanoma by activating the mTOR signaling 12. In pancreatic cancer, cell-intrinsic PD-1 promoted tumor growth through the Hippo signaling pathway outside the immune system 4, 20. However, Du et al found that cell-intrinsic PD-1 was presented in NSCLC as a tumor suppressor 41. In current study, we proved that patients with positive expression of PD-1 in NPC tissues had shorter survival time, which is consistent with findings in most malignancies. We also demonstrated that the positive expression rate of cancer cell-intrinsic PD-1 in metastatic NPC lesions was obviously higher than that in primary lesions. Although we haven't discovered statistical difference of the expression of PD-1 in primary NPC with or without lymph node metastasis, it was worth noting that the immune morphology of NPC cells altered during distant metastasis. Consequently, cell-intrinsic PD-1 might, we speculated, participate in the distant metastases of NPC. However, the number of matched primary/metastatic and lymph node metastases is limited, so further experimentation with larger size is needed to confirm the association between PD-1 expression and the NPC metastasis.

The PI3K signaling pathway participates PD-L1 expression regulation. S6 is one of the downstream targets in the PI3K/AKT/mTOR signaling pathway. Overexpression of p-S6 results in mTOR signaling pathway dysregulation 42-44. PD-1 promoted S6 phosphorylation and tumor proliferation in melanoma 12. Our results showed that positive p-S6 expression shortened overall survival in NPC patients, and there was an association between p-S6 expression and PD-L1 and PD-1 expression. At the same time, the p-S6 positive rate in patients with co-expression of PD-L1 and PD-1 was significantly higher than other patients. Hence, we speculated that PD-1 might also play an encouraging role in S6 phosphorylation in NPC. In conclusion, expression of cancer cell-intrinsic PD-1, PD-L1, co-expression of PD-L1 and PD-1 and p-S6 are correlational, especially between PD-1 and combined PD-L1 and PD-1 expression. However, these need to be further verified by *in vitro* and *in vivo* study in the future experiments.

In summary, in our study, there was significantly higher positive expression of cancer cell-intrinsic PD-1, PD-L1, p-S6 and co-expression of PD-1 and PD-L1 in NPC. Furthermore, PD-1 might also be involved in the metastatic spread of NPC. Positive PD-L1 expression associated with PD-1 and p-S6 expression, high expression of PD-L1 and p-S6 could serve as valuable independent prognostic biomarkers for NPC patients.

## Conclusion

Positive expression of PD-L1 associates with expression of cancer cell-intrinsic PD-1 and p-S6, PD-L1 might serve as a good prognostic biomarker and p-S6 could be a valuable independent poor prognostic biomarker for NPC patients.

## Supplementary Material

Supplementary table S1.

## Figures and Tables

**Table 1:** Association between expression of PD-L1, PD-1 and p-S6 proteins and clinicopathological features of NPC (n=281)

Parameter (n)	PD-L1		*P*-values	PD-1		*P*-values	p-S6		*P*-values	PD-1/PD-L1^#^		*P*-values
	P (%)	N (%)		P (%)	N (%)		P (%)	N (%)		H (%)	L (%)	
**Gender**												
Male (203)	130 (64.0)	73 (36.0)	0.326	46 (22.7)	157 (77.3)	0.697	181 (89.2)	22 (10.8)	0.110	38 (18.7)	165 (81.3)	0.513
Female (78)	45 (57.7)	33 (42.3)		16 (20.5)	62 (79.5)		64 (82.1)	14 (17.9)		12 (15.4)	66 (84.6)	
**Age (yr)^##^**												
<50 (146)	88 (60.3)	58 (39.7)	0.471	29 (19.9)	117 (80.1)	0.355	126 (86.3)	20 (13.7)	0.644	27 (18.5)	119 (81.5)	0.750
≥50 (135)	87 (64.4)	48 (35.6)		33 (24.4)	102 (75.6)		119 (88.1)	16 (11.9)		23 (17.0)	112 (83.0)	
**Histological type**												
DNPC (12)	7 (58.3)	5 (41.7)	0.773	2 (16.7)	10 (83.3)	0.645	9 (75.0)	3 (25.0)	0.197	2 (16.7)	10 (83.3)	0.917
UDNPC (269)	168 (62.5)	101 (37.5)		60 (22.3)	209 (77.7)		236 (87.7)	33 (12.3)		48 (17.8)	221 (82.2)	
**Clinical T category**												
T1 (25)	7 (28.0)	18 (72.0)	0.002**	5 (20.0)	20 (80.0)	0.921	19 (76.0)	6 (24.0)	0.357	2 (8.0)	23 (92.0)	0.317
T2 (103)	65 (63.1)	38 (36.9)		23 (22.3)	80 (77.7)		91 (88.3)	12 (11.7)		16 (15.5)	87 (84.5)	
T3 (78)	51 (65.4)	27 (34.6)		19 (24.4)	59 (75.6)		68 (87.2)	10 (12.8)		18 (23.1)	60 (76.9)	
T4 (75)	52 (69.3)	23 (30.7)		15 (20.0)	60 (80.0)		67 (89.3)	8 (10.7)		14 (18.7)	61 (81.3)	
**Clinical N category**												
N0 (46)	29 (63.0)	17 (37.0)	0.015*	14 (30.4)	32 (60.6)	0.416	43 (93.5)	3 (6.5)	0.180	10 (21.7)	36 (78.3)	0.729
N1 (82)	49 (59.8)	33 (40.2)		16 (19.5)	66 (80.5)		72 (87.8)	10 (12.2)		13 (15.9)	69 (84.1)	
N2 (122)	85 (69.7)	37 (30.3)		24 (19.7)	98 (80.3)		101 (82.8)	21 (17.2)		23 (18.9)	99 (81.1)	
N3 (31)	12 (38.7)	19 (61.3)		8 (25.8)	23 (74.2)		29 (93.5)	2 (6.5)		4 (12.9)	27 (87.1)	
**Clinical M category**												
M0 (272)	171 (62.9)	101 (37.1)	0.262	59 (21.7)	213 (78.3)	0.407	237 (87.1)	35 (12.9)	0.887	48 (17.6)	224 (82.4)	0.7249
M1 (9)	4 (44.4)	5 (55.6)		3 (33.3)	6 (66.7)		8 (88.9)	1 (11.1)		2 (22.2)	7 (77.8)	
**Clinical stage**												
I and II (n=57)	34 (59.6)	23 (40.4)	0.647	10 (17.5)	47 (82.5)	0.357	52 (91.2)	5 (8.8)	0.307	7 (12.3)	50 (87.7)	0.223
III and IV (224)	141 (62.9)	83 (37.1)		52 (23.2)	172 (76.8)		193 (86.2)	31 (13.8)		43 (19.2)	181 (80.8)	
**Lymph node status**												
LNM (235)	146 (62.1)	89 (37.9)	0.907	48 (20.4)	187 (79.6)	0.134	202 (86.0)	33 (14.0)	0.163	40 (17.0)	195 (83.0)	0.444
No LNM (46)	29 (63.0)	17 (37.0)		14 (30.4)	32 (69.6)		43 (93.5)	3 (6.5)		10 (21.7)	36 (78.3)	

Abbreviations: NPC: nasopharyngeal carcinoma; DNPC: differentiated non-keratinizing nasopharyngeal carcinoma; UDNPC: undifferentiated non-keratinizing nasopharyngeal carcinoma; LNM: lymph node metastasis; *N* negative; *P* positive.^#^Co-expression of PD-1 and PD-L1; ^##^the average age of all subjects was 49.8 years;*Correlation is significant at the *P*<0.05 level (two tailed). **Correlation is significant at the *P*<0.01 level (two tailed).

**Table 2:** The pairwise correlation between expression of PD-L1, PD-1 and p-S6 proteins in the 281 cases of NPC

	PD-L1	PD-1	p-S6	PD-1/PD-L1^#^
**PD-L1**				
Values	-	0.219	0.273	0.366
Significant		0.000**	0.000**	0.000**
**PD-1**				
Values	-	-	0.127	0.885
Significant			0.033*	0.000**
**p-S6**				
Values	-	-	-	0.153
Significant				0.010*

Values are Spearman's rank correlation coefficient.^#^ Co-expression of PD-1 and PD-1; *Correlation is significant at the *P*<0.05 level (two tailed); **Correlation is significant at the *P*<0.05 level (two tailed).

**Table 3:** Summary of multivariate Cox proportional hazard regression analysis used to evaluate overall survival in 281 cases of NPC patients

Parameter	SE	Wald	Significance	Exp(B)	95.0% CI for Exp(B)	
					Lower	Upper
Gender	0.305	0.626	0.429	0.785	0.432	1.429
Age	0.251	2.736	0.098	1.516	0.926	2.481
Histological type	1.017	0.759	0.384	0.412	0.056	3.024
LNM status	0.412	8.504	0.004**	3.329	1.483	7.470
Clinical T category	0.169	1.090	0.297	1.193	0.856	1.662
Clinical N category	0.167	12.792	0.000**	1.817	1.310	2.521
Clinical M category	0.452	16.961	0.000**	6.426	2.651	15.575
Clinical stages	0.176	7.830	0.005**	1.637	1.159	2.312
PD-L1	0.301	9.434	0.002**	0.397	0.220	0.716
PD-1	0.501	1.626	0.202	1.895	0.709	5.064
p-S6	0.481	8.665	0.003**	4.115	1.604	10.556
PD-L1/PD-1	0.607	0.170	0.680	1.284	0.391	4.217

**Abbreviations:** SE, standard error (SE); Exp (B), exponentiation of the B coefficient; CI, confidence interval; LNM: lymph node metastasis; NPC: nasopharyngeal carcinoma. Note: multivariate analysis of Cox proportional hazard regression, *Correlation is significant at the *P*<0.05 level (two tailed). **Correlation is significant at the *P*<0.01 level (two tailed).

## Abbreviations

PD-L1: programmed cell death ligand 1
PD-1: programmed death 1
NPC: nasopharyngeal carcinoma
IFNγ: interferon gamma
