# Supplementary material for: Expression of cancer cell-intrinsic PD-1 associates with PD-L1 and p-S6 and predicts a good prognosis in nasopharyngeal carcinoma
Source: J Cancer. 2021 Aug 24;12(20):6118–25. doi: 10.7150/jca.60739 (PMC8425204; doi:10.7150/jca.60739)
Supplement: Supplementary file 1 — Supplementary table S1. [file jcav12p6118s1.pdf]

Table S1. Clinicopathological features of patients with NPC and non-cancerous nasopharyngeal epithelia

Patients features	Primary NPC	Primary/metastases NPC	Non-cancerous nasopharyngeal epithelia
**Gender**			
Male	203(72.2%)	19(79.2%)	32(62.7%)
Female	78(27.8%)	5(20.8%)	19(37.3%)
**Age (yr)**			
＜50	146(52.0％)	15(62.5%)	31(60.8%)
≥50	135(48.0%)	9(37.5%)	20(39.2%)
**Clinical T category**			
T1	25(8.9%)		
T2	103(36.7%)		
T3	78(27.8%)		
T4	75(26.7%)		
**Clinical N category**			
N0	46(16.4%)		
N1	82(29.2%)		
N2	122(43.4%)		
N3	31(11.0%)		
**Clinical M category**			
M0	272(96.8%)		
M1	9(3.2%)		
**Clinical stage**			
Ⅰ	2(0.7%)		
Ⅱ	55(19.6%)		
Ⅲ	131(46.6%)		
Ⅳ	93((33.1%)		
**Histological type**			
DNC	12(4.3%)		
UDNC	269(95.7%)		
**LNM status**			
LNM	235(83.6%)		
No LNM	46(16.4%)		
**Survival situation**			
Alive	210(74.7%)		
Dead	71(25.3%)		
**EBV status**			
EBER positive	279(99.28)	24(100)	1(1.96)
EBER negative	2(0.72)	0(0.0)	50(99.04)
**Total Number**	281	24	51

Abbreviations: NPC nasopharyngeal carcinoma; DNPC differentiated non-keratinizing nasopharyngeal carcinoma; UDNPC undifferentiated non-keratinizing nasopharyngeal carcinoma; LNM: lymph node metastasis; EBV: Epstein–Barr virus; EBER: Epstein–Barr virus encoded RNAs
